# Supplementary material for: An environmental justice analysis of air pollution emissions in the United States from 1970 to 2010
Source: Nat Commun. 2024 Jan 17;15:268. doi: 10.1038/s41467-023-43492-9 (PMC10794183; doi:10.1038/s41467-023-43492-9)
Supplement: Supplementary file 1 — Supplementary Information [file 41467_2023_43492_MOESM1_ESM.pdf]

# An Environmental Justice Analysis of Air Pollution Emissions in the United States from 1970 to 2010

Yanelli Nunez *et al.*

This PDF file includes:

Supplementary Table 1. Effect estimates for linear associations
Supplementary Table 2. Summary of models in the main analysis
Supplementary Table 3. Confounding by socioeconomic status sensitivity analysis summary of models
Supplementary Table 4. Regional sensitivity analysis summary of models
Supplementary Fig. 1. Spearman correlation coefficient among demographic variables
Supplementary Fig. 2. Trends in emission fluxes from 1970 to 2010
Supplementary Fig. 3. Sensitivity analysis: confounding by economic status
Supplementary Fig. 4. Transportation sector regional analysis
Supplementary Fig. 5. Industry sector regional analysis
Supplementary Fig. 6. EPA geographic regions
Supplementary Fig. 7. Urbanicity maps for the years 1970 and 2000

**Supplementary Table 1. Effect estimates for linear associations**. Estimates represent percentage point increases in the relative change of emissions per a 10 percentage point increase in county-level White, Black, Asian, American Indian, or Hispanic population. Effect estimates are presented only for the linear associations. For all results (linear and non-linear), see manuscript Figures 2 and 3.

| | Effect Estimate (95% CI) |
|---|---|
| **Industry: $SO_2$** | |
| % Black | -1.35 (-2.09, -0.60) |
| | |
| **Energy: $SO_2$** | |
| % Asian | -4.98 (-22.87, 12.90) |
| % Hispanic | 4.24 (-2.76, -11.24) |
| | |
| **Energy: $NO_x$** | |
| % White | -9.25 (-15.01, -3.48) |
| % Black | -17.43 (-23.88, -10.98) |
| % Asian | 18.52 (7.41, 29.63) |
| % American Indian | 11.78 (4.20, 19.38) |
| | |
| **Agriculture: $NH_3$** | |
| % American Indian | 0.33 (-0.02, 0.67) |
| | |
| **Transport: $NO_x$** | |
| % American Indian | -0.13 (-0.33, 0.06) |
| | |
| **Residential: OC** | |
| % Black | -0.37 (-0.54, -0.19) |

**Supplementary Table 2. Summary of models in the main analysis**. Each air pollution emissions sector included eight models (one for each socioeconomic/racial/ethnic variable of interest). In total, the main analysis included 48 independent models.

| | Outcome (% Emissions Change) | Predictor Variable of Interest | Covariates |
|---|---|---|---|
| 1 | | % White & %Black | Population density, urbanicity and year as categorical variables, and % White population |
| 2 | | % Asian | |
| 3 | | % American Indian | |
| 4 | Industry $SO_2$ | % Hispanic | |
| 5 | N = 12,404 counties | % Poverty | Population density, urbanicity and year as categorical variables |
| 6 | | % Unemployment | |
| 7 | | Median Family Income | |
| 8 | | Median Property Value | |
| 1 | | % White & %Black | Population density, urbanicity and year as categorical variables, and % White population |
| 2 | | % Asian | |
| 3 | | % American Indian | |
| 4 | Energy $SO_2$ | % Hispanic | |
| 5 | N = 11,788 counties | % Poverty | Population density, urbanicity and year as categorical variables |
| 6 | | % Unemployment | |
| 7 | | Median Family Income | |
| 8 | | Median Property Value | |
| 1 | | % White & %Black | Population density, urbanicity and year as categorical variables, and % White population |
| 2 | | % Asian | |
| 3 | | % American Indian | |
| 4 | Energy $NO_x$ | % Hispanic | |
| 5 | N = 11,788 counties | % Poverty | Population density, urbanicity and year as categorical variables |
| 6 | | % Unemployment | |
| 7 | | Median Family Income | |
| 8 | | Median Property Value | |
| 1 | | % White & %Black | Population density, urbanicity and year as categorical variables, and % White population |
| 2 | | % Asian | |
| 3 | | % American Indian | |
| 4 | Agriculture $NH_3$ | % Hispanic | |
| 5 | N = 12,409 | % Poverty | Population density, urbanicity and year as categorical variables |
| 6 | | % Unemployment | |
| 7 | | Median Family Income | |
| 8 | | Median Property Value | |
| 1 | | % White & %Black | Population density, urbanicity and year as categorical variables, and % White population |
| 2 | | % Asian | |
| 3 | | % American Indian | |
| 4 | Transport $NO_x$ | % Hispanic | |
| 5 | N = 12,409 | % Poverty | Population density, urbanicity and year as categorical variables |
| 6 | | % Unemployment | |
| 7 | | Median Family Income | |
| 8 | | Median Property Value | |
| 1 | | % White & %Black | Population density, urbanicity and year as categorical variables, and % White population |
| 2 | | % Asian | |
| 3 | | % American Indian | |
| 4 | Residential Organic Carbon | % Hispanic | |
| 5 | N = 12,409 | % Poverty | Population density, urbanicity and year as categorical variables |
| 6 | | % Unemployment | |
| 7 | | Median Family Income | |
| 8 | | Median Property Value | |

**Supplementary Table 3. Summary of models in the confounding by socioeconomic status sensitivity analysis**. We ran the race/ethnicity models adjusting for socioeconomic status. In total, this sensitivity analysis included 24 separate models.

| | Outcome (% Emissions Change) | Predictor Variable of Interest | Covariates |
|---|---|---|---|
| 1 | Industry $SO_2$ N = 12,404 counties | % White & %Black | Population density, urbanicity and year as categorical variables, median family income, % poverty, % unemployment, median property value |
| 2<br>3<br>4 | | % Asian<br>% American Indian<br>% Hispanic | % White, population density, urbanicity and year as categorical variables, median family income, % poverty, % unemployment, median property value |
| 1 | Energy $SO_2$ N = 11,788 counties | % White & %Black | Population density, urbanicity and year as categorical variables, median family income, % poverty, % unemployment, median property value |
| 2<br>3<br>4 | | % Asian<br>% American Indian<br>% Hispanic | % White, population density, urbanicity and year as categorical variables, median family income, % poverty, % unemployment, median property value |
| 1 | Energy $NO_x$ N = 11,788 counties | % White & %Black | Population density, urbanicity and year as categorical variables, median family income, % poverty, % unemployment, median property value |
| 2<br>3<br>4 | | % Asian<br>% American Indian<br>% Hispanic | % White, population density, urbanicity and year as categorical variables, median family income, % poverty, % unemployment, median property value |
| 1 | Agriculture $NH_3$ N = 12,409 | % White & %Black | Population density, urbanicity and year as categorical variables, median family income, % poverty, % unemployment, median property value |
| 2<br>3<br>4 | | % Asian<br>% American Indian<br>% Hispanic | % White, population density, urbanicity and year as categorical variables, median family income, % poverty, % unemployment, median property value |
| 1 | Transport $NO_x$ N = 12,409 | % White & %Black | Population density, urbanicity and year as categorical variables, median family income, % poverty, % unemployment, median property value |
| 2<br>3<br>4 | | % Asian<br>% American Indian<br>% Hispanic | % White, population density, urbanicity and year as categorical variables, median family income, % poverty, % unemployment, median property value |
| 1 | Residential Organic Carbon N = 12,409 | % White & %Black | Population density, urbanicity and year as categorical variables, median family income, % poverty, % unemployment, median property value |
| 2<br>3<br>4 | | % Asian<br>% American Indian<br>% Hispanic | % White, population density, urbanicity and year as categorical variables, median family income, % poverty, % unemployment, median property value |

**Supplementary Table 4. Summary of models in the regional sensitivity analysis**. We focused on the industry and on-road transport sectors. We ran two models for each EPA region, one with % White and % Black population and one with median property value as the predictor variables of interest. All models were adjusted for the same set of confounders. In total, the regional sensitivity analysis consisted of 32 separate models.

| EPA Region | N = # counties | Outcome (% emissions change) | Predictor of Interest | Covariates |
|---|---|---|---|---|
| 1-3 | 1,726 | Industry $SO_2$ | % White & %Black<br>Median Property Value | Population density, urbanicity and year as a categorical variable |
| 4 | 2,944 | | % White & %Black<br>Median Property Value | |
| 5 | 2,090 | | % White & %Black<br>Median Property Value | |
| 6 | 2,010 | | % White & %Black<br>Median Property Value | |
| 7 | 1,647 | | % White & %Black<br>Median Property Value | |
| 8 | 1,157 | | % White & %Black<br>Median Property Value | |
| 9 | 358 | | % White & %Black<br>Median Property Value | |
| 10 | 472 | | % White & %Black<br>Median Property Value | |
| 1-3 | 1,726 | Transport $NO_x$ | % White & %Black<br>Median Property Value | Population density, urbanicity and year as a categorical variable |
| 4 | 2,944 | | % White & %Black<br>Median Property Value | |
| 5 | 2,091 | | % White & %Black<br>Median Property Value | |
| 6 | 2,010 | | % White & %Black<br>Median Property Value | |
| 7 | 1,648 | | % White & %Black<br>Median Property Value | |
| 8 | 1,160 | | % White & %Black<br>Median Property Value | |
| 9 | 358 | | % White & %Black<br>Median Property Value | |
| 10 | 472 | | % White & %Black<br>Median Property Value | |

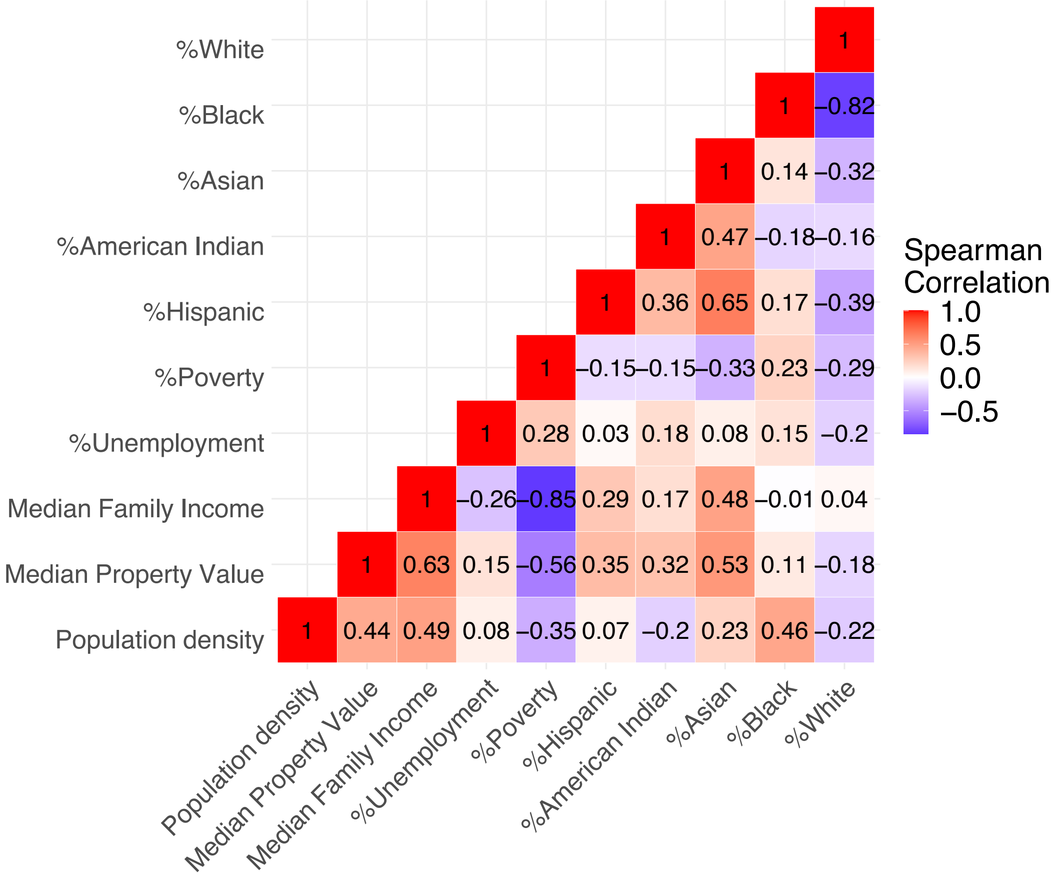


**Supplementary Fig. 1. Spearman correlation coefficients for the demographic variables**. Coefficients were estimated from scaled variables using all observations from 1970 to 2000.

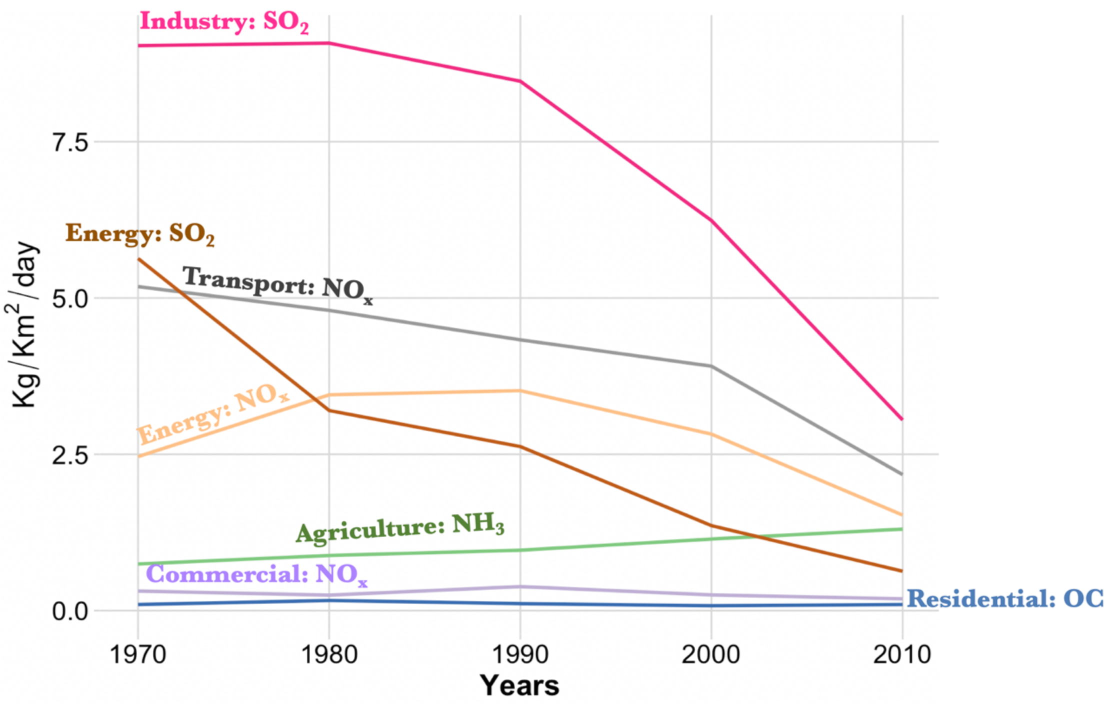


**Supplementary Fig. 2.** Trends in county-level mean air pollution emissions from 1970 to 2010

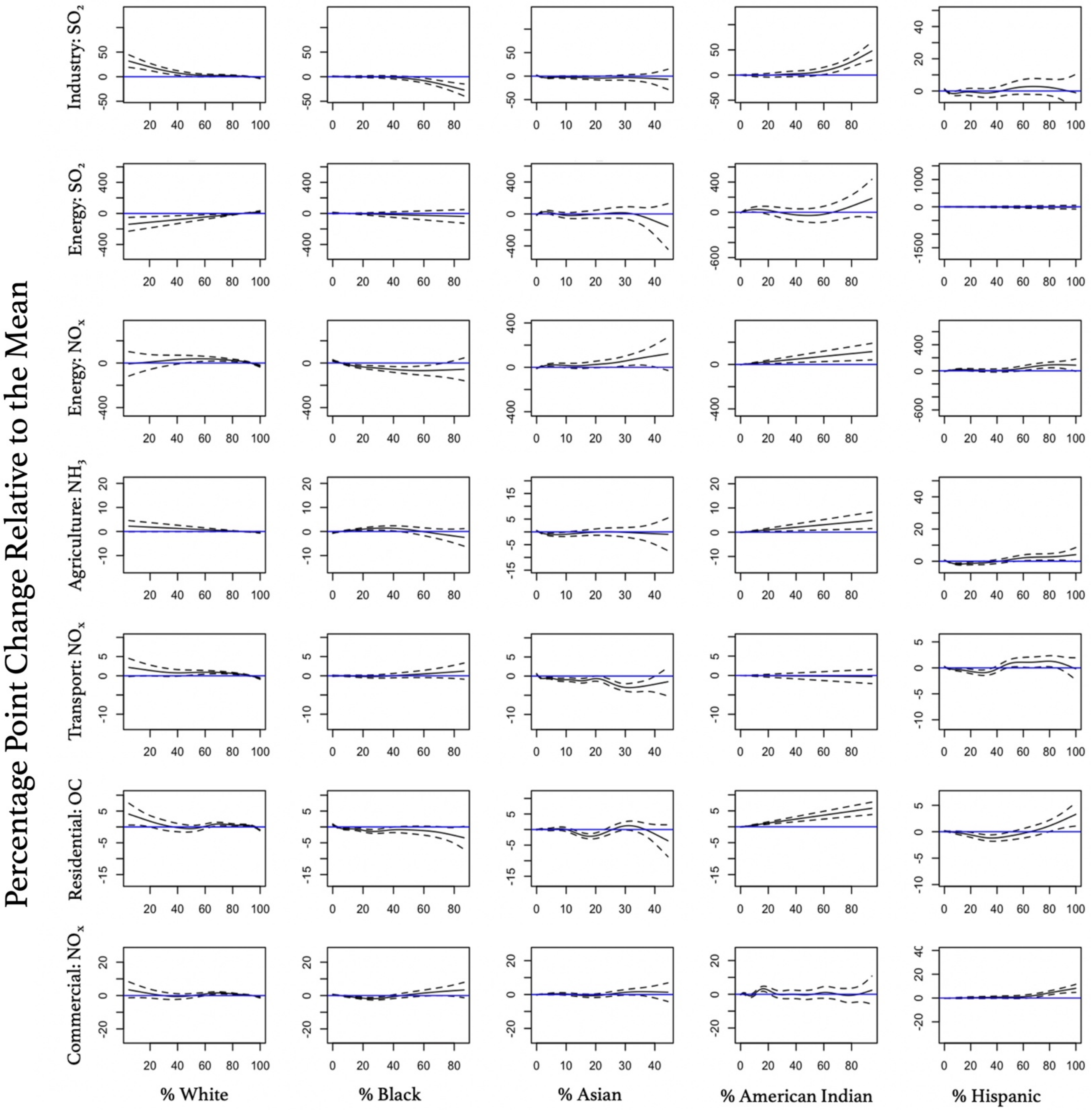


**Supplementary Fig. 3. Sensitivity analysis: confounding by socioeconomic status**. The x-axes are the county-level White, Black, Asian, American Indian, or Hispanic percentage population, and the y-axes correspond to percentage point change at the relative emissions in reference to the mean demographic variable. Models were adjusted for population density, urbanicity, EPA geographic region, year, percent unemployment, percent poverty, median family income, and median property value. The dashed lines on the curve are the 95% confidence interval (CI).

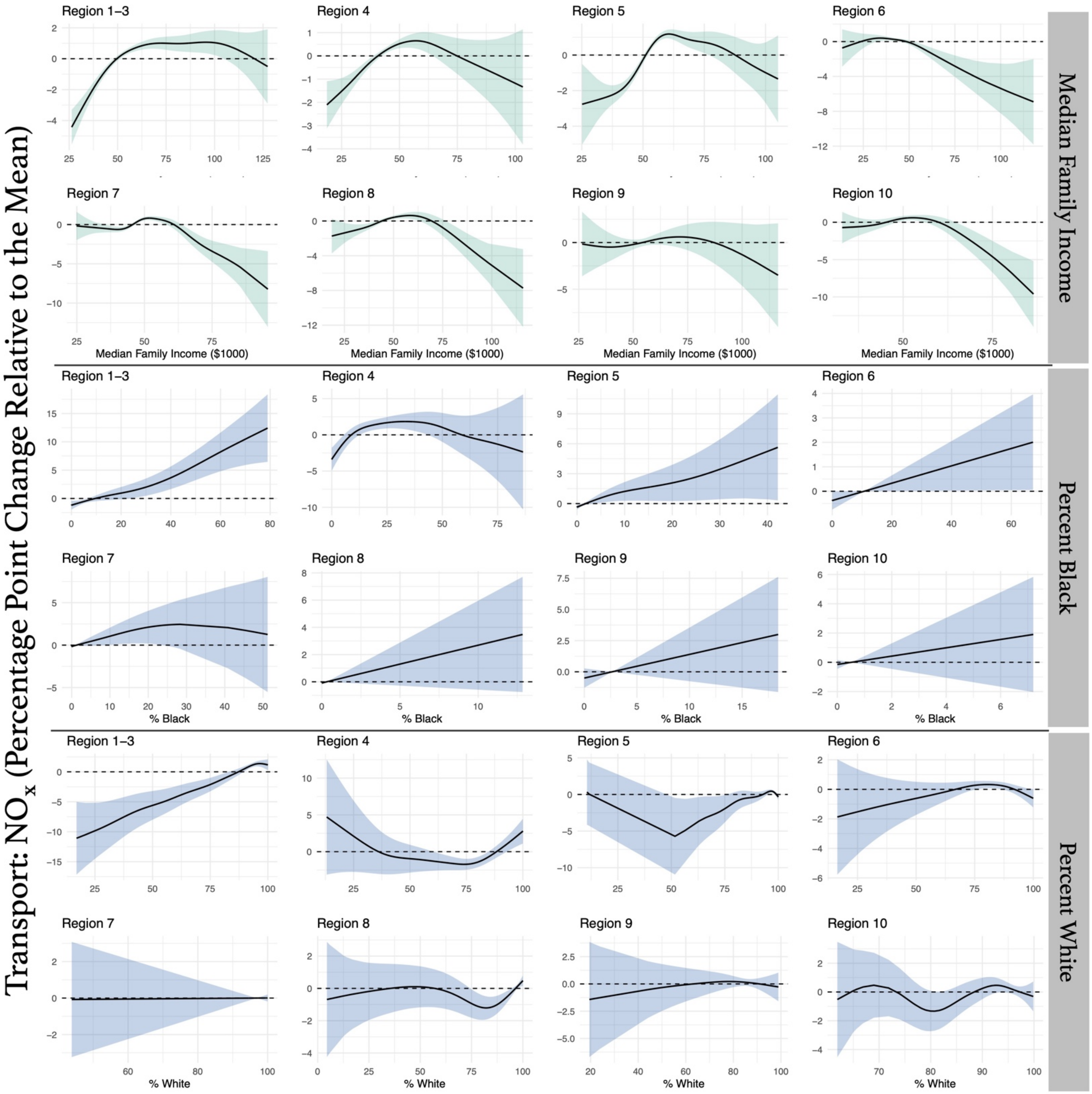


**Supplementary Fig. 4. Sensitivity analysis: regional analysis for transport $NO_x$**. The x-axes are the county-level percent population White, percent population Black, or median family income. The y-axes correspond to percentage point change at the relative emissions in reference to the mean demographic variable. Models were adjusted for population density, urbanicity, and year. The shaded areas represent the 95% confidence interval. The regions correspond to the EPA geographic regions (Supplementary Fig.6).

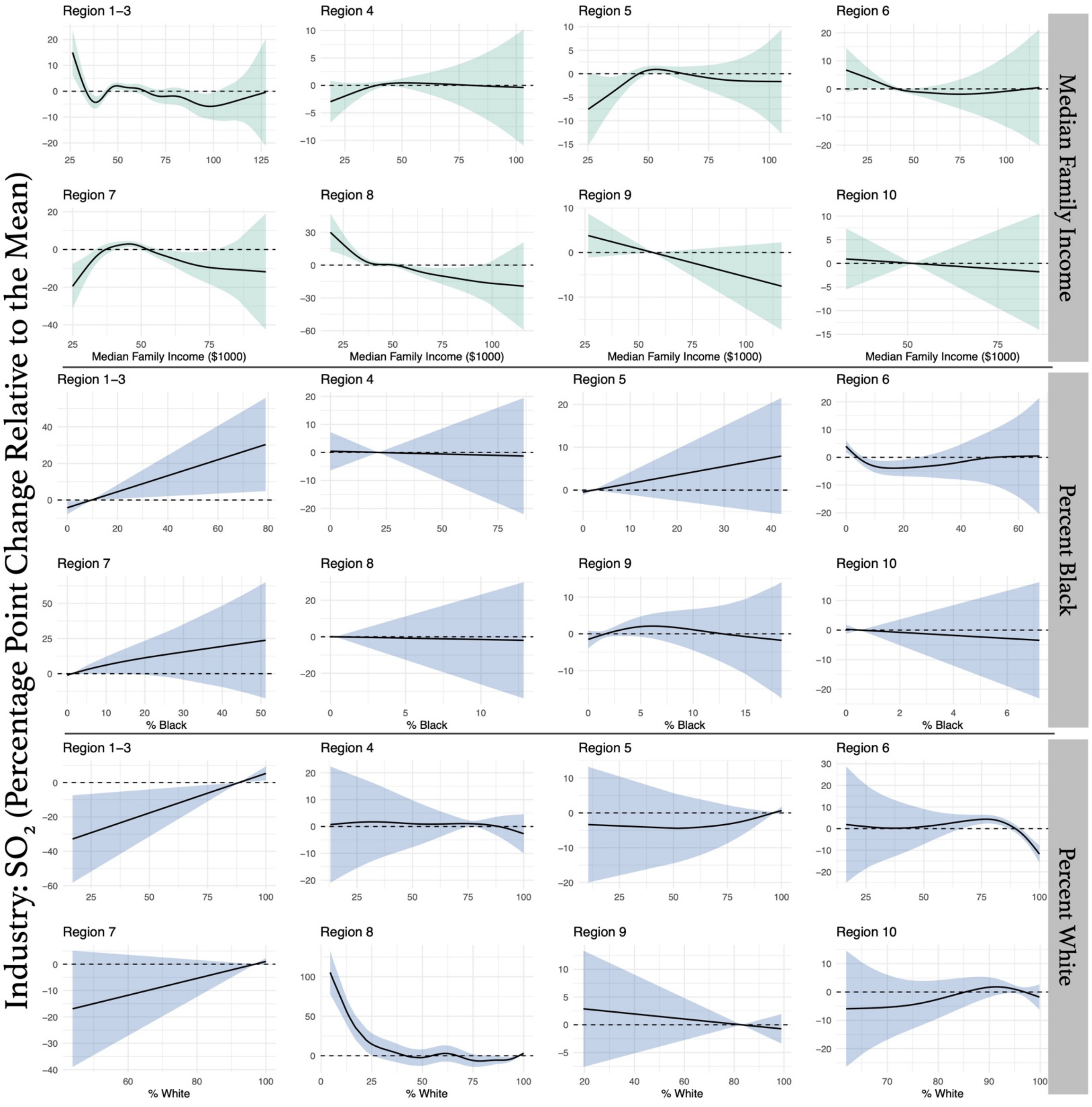


**Supplementary Fig. 5: Sensitivity analysis: regional analysis for industry $SO_2$.** The x-axes are the county-level percent population White, percent population Black, or median family income, and the y-axes and the y-axes correspond to percentage point change at the relative emissions in reference to the mean demographic variable. Models were adjusted for population density, urbanicity, and year. The shaded areas represent the 95% confidence interval. The regions correspond to the EPA geographic regions (Supplementary Fig. 6).

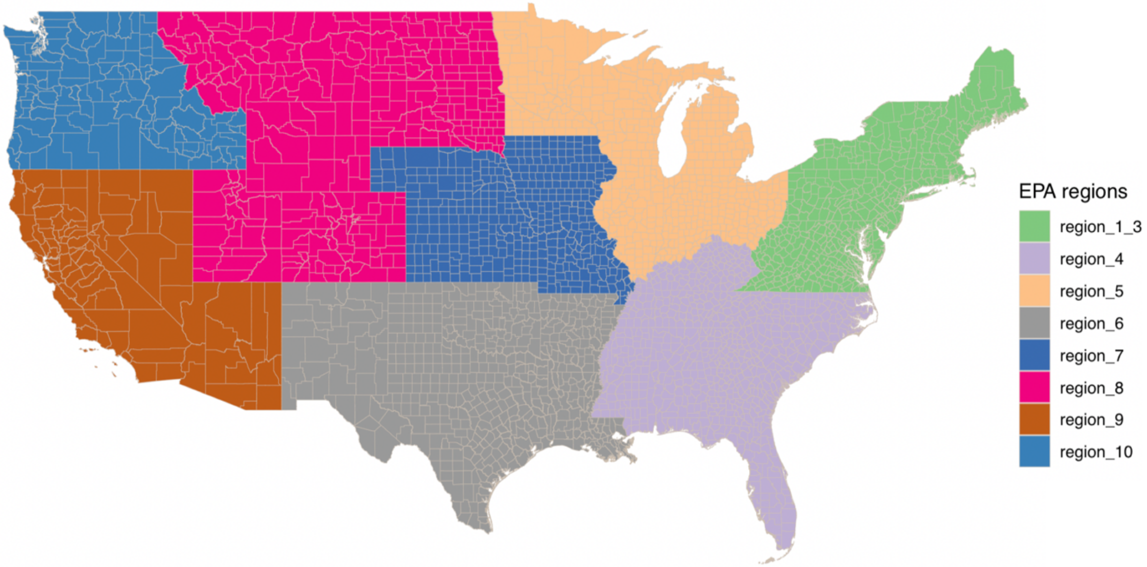


**Supplementary Fig. 6. EPA geographic regions**. The regions were included as a categorical covariate in all analyses. This figure used county spatial shape files obtained from NHGIS.

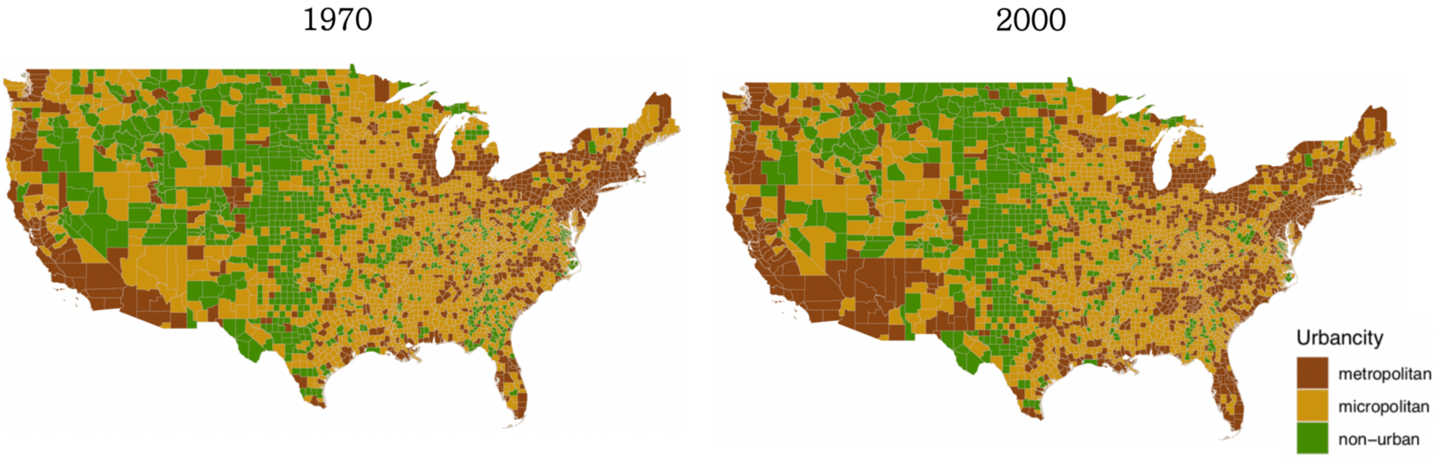


**Supplementary Fig. 7. Maps show counties by urbanicity level in 1970 and 2000**. The urbanicity categories are based on population: metropolitan (population ≥ 50,000), micropolitan (50,000 > population ≥ 10,000), and non-urban (population < 10,000). All analyses included urbanicity as a categorical variable. This figure used county spatial shape files obtained from NHGIS.
