# Supplementary material for: An environmental justice analysis of air pollution emissions in the United States from 1970 to 2010
Source: Nat Commun. 2024 Jan 17;15:268. doi: 10.1038/s41467-023-43492-9 (PMC10794183; doi:10.1038/s41467-023-43492-9)
Supplement: Supplementary file 3 — Reporting Summary [file 41467_2023_43492_MOESM3_ESM.pdf]

nature portfolio

Corresponding author(s): Yanelli Nunez

Last updated by author(s): Oct 30, 2023

Reporting Summary

Nature Portfolio wishes to improve the reproducibility of the work that we publish. This form provides structure for consistency and transparency in reporting. For further information on Nature Portfolio policies, see our Editorial Policies and the Editorial Policy Checklist.

Statistics

For all statistical analyses, confirm that the following items are present in the figure legend, table legend, main text, or Methods section.

n/a	Confirmed	
☐	☒	The exact sample size (*n*) for each experimental group/condition, given as a discrete number and unit of measurement
☒	☐	A statement on whether measurements were taken from distinct samples or whether the same sample was measured repeatedly
☒	☐	The statistical test(s) used AND whether they are one- or two-sided<br>*Only common tests should be described solely by name; describe more complex techniques in the Methods section.*
☐	☒	A description of all covariates tested
☒	☐	A description of any assumptions or corrections, such as tests of normality and adjustment for multiple comparisons
☐	☒	A full description of the statistical parameters including central tendency (e.g. means) or other basic estimates (e.g. regression coefficient) AND variation (e.g. standard deviation) or associated estimates of uncertainty (e.g. confidence intervals)
☒	☐	For null hypothesis testing, the test statistic (e.g. *F*, *t*, *r*) with confidence intervals, effect sizes, degrees of freedom and *P* value noted<br>*Give P values as exact values whenever suitable.*
☒	☐	For Bayesian analysis, information on the choice of priors and Markov chain Monte Carlo settings
☐	☒	For hierarchical and complex designs, identification of the appropriate level for tests and full reporting of outcomes
☐	☒	Estimates of effect sizes (e.g. Cohen's *d*, Pearson's *r*), indicating how they were calculated

Our web collection on statistics for biologists contains articles on many of the points above.

Software and code

Policy information about availability of computer code

Data collection	No software was use for data collection
Data analysis	All analyses were performed using R version 4.1.2 running under macOS Monterey 12.3. Primarily, the package tidyverse version 2.0.0 was used for the cleaning and wrangling of demo-graphic data; package RNetCDF version 2.8-1 and PCICt version 0.5-4.4 for the procession of emissions data; function gamm4 from the package gamm4 version 0.2-6 was used for all modeling analyses; and ggplot2 version 3.4.2 was used for data visualizations. For a complete list of the packages and code used in the analysis, see https://github.com/yanellinunez/USA_emissions_code.

For manuscripts utilizing custom algorithms or software that are central to the research but not yet described in published literature, software must be made available to editors and reviewers. We strongly encourage code deposition in a community repository (e.g. GitHub). See the Nature Portfolio guidelines for submitting code & software for further information.

Data

Policy information about availability of data

All manuscripts must include a data availability statement. This statement should provide the following information, where applicable:

- Accession codes, unique identifiers, or web links for publicly available datasets
- A description of any restrictions on data availability
- For clinical datasets or third party data, please ensure that the statement adheres to our policy

The raw emissions data used in this study can be accessed at https://zenodo.org/record/3754964#.Y-bEE-zMLOS and the raw demographic data via https://data.census.gov/ or NHGIS.org. The processed emissions and demographic data have been deposited in a publicly available GitHub repository [https://github.com/yanellinunez/USA_emissions_code].

Research involving human participants, their data, or biological material

Policy information about studies with human participants or human data. See also policy information about sex, gender (identity/presentation), and sexual orientation and race, ethnicity and racism.

Reporting on sex and gender	N/A
Reporting on race, ethnicity, or other socially relevant groupings	N/A
Population characteristics	N/A
Recruitment	N/A
Ethics oversight	N/A

Note that full information on the approval of the study protocol must also be provided in the manuscript.

Field-specific reporting

Please select the one below that is the best fit for your research. If you are not sure, read the appropriate sections before making your selection.

☐ Life sciences ☒ Behavioural & social sciences ☐ Ecological, evolutionary & environmental sciences

For a reference copy of the document with all sections, see nature.com/documents/nr-reporting-summary-flat.pdf

Behavioural & social sciences study design

All studies must disclose on these points even when the disclosure is negative.

Study description	Over the last decades, air pollution emissions have decreased substantially; however, inequi-ties in air pollution persist. We evaluate county-level racial/ethnic and socioeconomic dispari-ties in emissions changes from six air pollution source sectors (industry [SO2], energy [SO2, NOx], agriculture [NH3], commercial [NOx], residential [particulate organic carbon], and on-road transportation [NOx]) in the contiguous United States during the 40 years following the Clean Air Act (CAA) enactment (1970-2010). We calculate relative emission changes and ex-amine the differential changes given county demographics using hierarchical nested models. The results show racial/ethnic disparities, particularly in the industry and energy generation source sectors. We also found that median family income is a driver of variation in relative emissions changes in all sectors—counties with median family income >$75K generally experi-ence larger relative declines in industry, energy, transportation, residential, and commercial-related emissions. Emissions from most air pollution source sectors have, on a national level, decreased following the United States CAA. In this work, we show that the relative reductions in emissions varied across racial/ethnic and socioeconomic groups.
Research sample	The total sample numbers were as follows: the industry source sector analysis included 12404 counties, the energy source sector included 11788 counties, and the residential, transportation, agriculture, and commercial source sector analyses included 12409 counties.
Sampling strategy	N/A
Data collection	N/A
Timing	Decennial data from 1970-2010 at the county level
Data exclusions	From the analysis, we excluded Yellowstone National Park County (average population = 130 individuals), which had a negative property value in the 1970 census data. We removed three counties (Broomfield, CO; Washabaugh, SD; and Nansemond, VA) that

existed for only one year during the study period; thus, we could not estimate a change in emissions. We also removed five small islands and coastal counties for which we could not estimate emissions (Nantucket, MA; Mathews, VA; San Juan, WA; Door, WI; Poquoson, VA). In 1970, the Census did not collect race/ethnicity and ethnicity data for DC or property value for Adam's County, WI; thus, these two counties only contributed to the analysis from 1980 to 2010. Additionally, estimating relative changes in energy and industrial emissions resulted in observations with high (i.e., emissions changed from a value close to zero to a large value) and infinite values (i.e., emissions changed from nonzero to zero), respectively. These extreme values affect the proper modeling of associations. Thus, we removed five counties with infinite values in the industry sector and excluded observations in the top 5 percentile in the energy sector.

Non-participation: N/A

Randomization: N/A

Reporting for specific materials, systems and methods

We require information from authors about some types of materials, experimental systems and methods used in many studies. Here, indicate whether each material, system or method listed is relevant to your study. If you are not sure if a list item applies to your research, read the appropriate section before selecting a response.

Materials & experimental systems

n/a	Involved in the study
☒	☐ Antibodies
☒	☐ Eukaryotic cell lines
☒	☐ Palaeontology and archaeology
☒	☐ Animals and other organisms
☒	☐ Clinical data
☒	☐ Dual use research of concern
☒	☐ Plants

Methods

n/a	Involved in the study
☒	☐ ChIP-seq
☒	☐ Flow cytometry
☒	☐ MRI-based neuroimaging

Plants

Seed stocks: N/A

Novel plant genotypes: N/A

Authentication: N/A
